# Survival Strategies of *Staphylococcus aureus*: Adaptive Regulation of the Anti-Restriction Gene *ardA*-H1 Under Stress Conditions

**DOI:** 10.3390/antibiotics13121131

**Published:** 2024-11-25

**Authors:** Flavia Costa Carvalho de Andrade, Mariana Fernandes Carvalho, Agnes Marie Sá Figueiredo

**Affiliations:** 1 Departamento de Microbiologia Médica, Universidade Federal do Rio de Janeiro, Rio de Janeiro 21941-902, Brazil; flaviacca@micro.ufrj.br (F.C.C.d.A.); marianafc@micro.ufrj.br (M.F.C.); 2 Programa de Pós-Graduação em Patologia, Faculdade de Medicina, Universidade Federal Fluminense, Niterói 24033-900, Brazil

**Keywords:** anti-restriction proteins, environmental stress, *ardA*, MRSA, *S. aureus*, horizontal gene transfer, HGT, mutation, acidic stress, iron deprivation, antibiotic stress

## Abstract

**Background/Objective**: The anti-restriction protein ArdA-H1, found in multiresistant *Staphylococcus aureus* (MRSA) strains from the ST239-SCC*mec*III lineage, inhibits restriction–modification systems, fostering horizontal gene transfer (HGT) and supporting genetic adaptability and resistance. This study investigates the regulatory mechanisms controlling *ardA-*H1 expression in *S. aureus* under various stress conditions, including acidic pH, iron limitation, and vancomycin exposure, and explores the roles of the Agr quorum sensing system. **Methods**: The expression of *ardA-*H1 was analyzed in *S. aureus* strains exposed to environmental stressors using real-time quantitative reverse transcription PCR. Comparisons were made between Agr-functional and Agr-deficient strains. In addition, Agr inhibition was achieved using a heterologous Agr autoinducing peptide. **Results**: The Agr system upregulated *ardA-*H1 expression in acidic and iron-limited conditions. However, vancomycin induced *ardA-*H1 activation specifically in the Agr-deficient strain GV69, indicating that an alternative regulatory pathway controls *ardA-*H1 expression in the absence of *a*gr. The vancomycin response in GV69 suggests that diminished quorum sensing may offer a survival advantage by promoting persistence and HGT-related adaptability. **Conclusion**: Overall, our findings provide new insights into the intricate relationships between quorum-sensing, stress responses, bacterial virulence, and genetic plasticity, enhancing our understanding of *S*. *aureus* adaptability in challenging environments.

## 1. Introduction

Horizontal gene transfer (HGT) is a crucial mechanism by which bacteria acquire new genes, including those for antimicrobial resistance [1]. Alongside DNA mutations, HGT drives the evolution of bacterial pathogenicity and antimicrobial resistance [2]. Additionally, the selective pressure imposed by the widespread use of antimicrobials can increase HGT rates [3]. HGT occurs in various environments, such as the gut microbiome [4], sewage treatment plants [5], and soil, where herbicides like glyphosate can induce gene transfer [1]. Bacterial biofilms are particularly significant hotspots for HGT due to their ability to promote bacterial cell aggregation, facilitate communication through quorum sensing (which regulates the mechanism of competence), and enhance DNA release and uptake [6].

It has been demonstrated that the induction of the SOS system by mitomycin C or ciprofloxacin enhances HGT by more than 300-fold in both *Escherichia coli* and *Vibrio cholerae* [7]. More recently, it was reported that not only ciprofloxacin but also antibiotics such as azithromycin and tetracycline, can increase HGT in *V. cholerae* [8]. Additionally, β-lactam antibiotics have been shown to boost SOS expression and enhance HGT through natural competence in bacteria [9]. Furthermore, factors that elevate oxidative stress, such as UV irradiation, have been found to increase conjugation rates in *E. coli* [10]. Gashgari et al. studied the impact of single and multiple environmental stressors on *Acinetobacter baylyi*. They found that these stressors, which can be mutagenic, induce the accumulation of reactive oxygen species (ROS) and/or affect cell membrane permeability, triggering a bacterial response aimed at repairing the resulting damage. This response leads to an additive effect on transformation frequency, highlighting the complex interplay between stress responses and genetic exchange mechanisms in bacteria [11].

Despite this, bacteria have developed mechanisms to defend against DNA invaders while maintaining a balance between evolution and genetic stability. One such mechanism is the restriction–modification (RM) system [12]. RM complexes serve as the first line of defense against exogenous DNA, degrading nucleic acids introduced by phages, plasmids, and other mobile genetic elements (MGE). These systems typically consist of a restriction enzyme, which recognizes and cleaves unmethylated DNA, and a methyltransferase domain that protects endogenous DNA by methylating specific adenine or cytosine residues [13,14].

Genes encoding anti-restriction (AR) proteins, when present in some bacterial hosts, are activated on specific occasions to either attenuate or inhibit RM systems, facilitating higher rates of HGT [15]. Examples of AR proteins include ArdA and ArdB in *E. coli* [16], KlcAHS in *Klebsiella pneumoniae* [17], and the Ocr in T7 phage [18]. Additionally, a study identified ArdA homologs (ArdA-H1 and ArdA-H2) in pathogenic Gram-positive bacteria, such as *Staphylococcus aureus*, *Enterococcus faecium*, and *Streptococcus agalactiae* [19]. The researchers revealed that the genomes of highly multidrug-resistant MRSA strains (with more than four resistant genes) from ST239 and ST398 were enriched with *ardA*-H1 and *ardA*-H2 genes, respectively, compared to more susceptible MRSA strains [19].

Despite growing evidence that AR proteins contribute to the accelerated evolution of antimicrobial resistance, to the best of our knowledge, most studies on *ardA* genes have primarily focused on their mechanisms of action and their implications for antimicrobial resistance [12,13,14,19]. However, there is a noticeable gap in research regarding the regulation of AR genes. This gap is likely due to the fact that the expression of *ardA* genes from bacterial chromosomes has only recently been discovered in *S. aureus* [19] and *Bifidobacterium bifidum* [20]. Indeed, mutation rates are known to increase when bacteria are exposed to stressors, including nutritional deprivation, oxidative and temperature stress, DNA damage, and antibiotic exposure [21,22]. However, to the best of our knowledge, the effect of stress conditions on the expression of *ardA* genes remains unclear. In our previous research, we demonstrated that *ardA*-H1 is expressed as double-stranded DNA in recipient bacteria, leading to a 3.67-fold increase in HGT in strains harboring *ardA*-H1, compared to isogenic strains lacking this gene [19]. In the current study, we investigated the hypothesis that certain environmental factors may modulate *ardA*-H1 expression, potentially influencing gene transfer dynamics. Our findings provide new insights into *ardA*-H1 expression and its regulatory mechanisms in *S. aureus*.

## 2. Results

### 2.1. ArdA-H1 Expression Markedly Increases During Stationary Growth Phase

**Figure 1:**
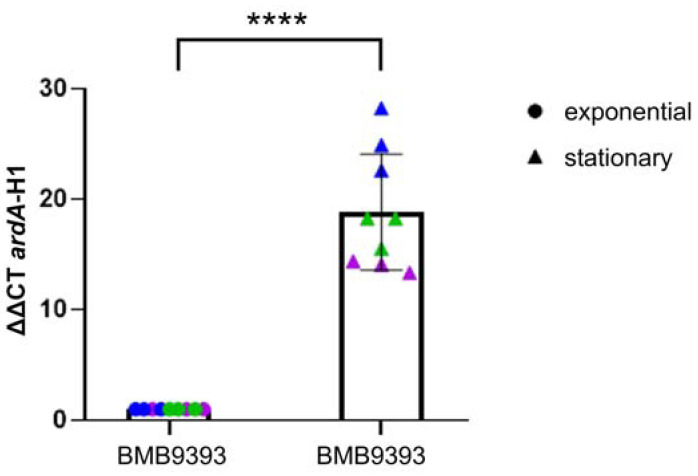
Relative quantitation (ΔΔCT) of *ardA*-H1 transcripts in strain BMB9393 during exponential and stationary growth phases. Individual data points from different replicates are shown in blue, green, and violet. For statistical calculation, data represent the mean of three independent biological experiments, each with three technical replicates (*n* = 3). A paired Student’s *t*-test was used to calculate *p*-value, with significance indicated as **** for *p* < 0.0001. The corresponding Bayes factor of 1.146 supports the alternative hypothesis over the null.

Quantitation of *ardA-*H1 transcripts in the BMB9393 strain—using real-time quantitative reverse transcription polymerase chain reaction (qRT-PCR)—revealed that *ardA*-H1 expression under normal growth conditions was 17.86 times higher in the stationary phase compared to the exponential phase when normalized to 1.0 (Figure 1). Consequently, all subsequent experiments were conducted during the stationary phase.

### 2.2. Effect of Agr in ardA-H1 Expression

**Figure 2:**
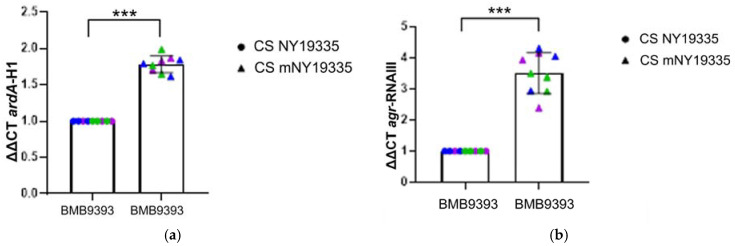

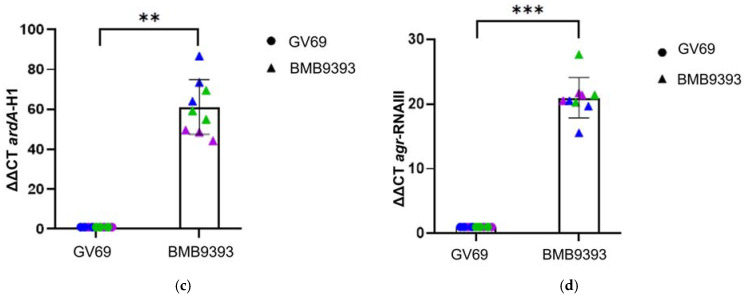
Relative quantitation (ΔΔCT) of *ardA*-H1 and *agr*-RNAIII transcripts. Expression levels of *ardA*-H1 (**a**) and *agr*-RNAIII (**b**) in strain BMB9393 treated with conditioned supernatant (CS), with (CS NY19335) and without (CS NYm19335) the heterologous type II Agr autoinducer peptide (AIP). The data used for statistical calculation represent the mean of three independent biological experiments, each with three technical replicates *(n* = 3). A paired Student’s *t*-test was used to calculate the *p*-value, with significance indicated as (**a**) *** for *p =* 0.0009 and (**b**) *** for *p =* 0.0002. The corresponding Bayes factors of 11.782 for *ardA*-H1 and 2.690 for *agr*-RNAIII support the alternative hypothesis over the null. Panels (**c**,**d**) show the quantification of *ardA*-H1 and *agr*-RNAIII transcripts, respectively, in strains BMB9393 and GV69. The data used for statistical calculation represent the mean of three independent biological experiments, each with three technical replicates (*n* = 3). An unpaired Student’s *t*-test was used to calculate *p*-value, with significance indicated as (**c**) ** for *p* = 0.0016 and (**d**) *** for *p* = 0.0001. The corresponding Bayes factor of 1.046 for *ardA*-H1 and 1.154 for *agr*-RNAIII, again supports the alternative hypothesis over the null. Individual data points from different replicates are shown in blue, green, and violet.

Given that the Agr system serves as a temporal global regulator in *S. aureus*, and that quorum sensing thresholds are established during the mid-log phase, the observed increase in *ardA*-H1 expression during the stationary phase, compared to the initial log phase, suggests that the main effector molecule of the Agr system, the RNAIII, regulates *ardA*-H1. To evaluate this hypothesis, we treated strain BMB9393 with the conditioned supernatant (CS) from strain NY19335, which contains a heterologous type II autoinducer peptide (AIP). This peptide competes with the homologous type I AIP of the strain BMB9393, thereby inhibiting its Agr system. In fact, our data revealed a 3.5-fold reduction in *rnaIII* expression in strain BMB9393, accompanied by an approximately 2.0-fold decrease in *ardA*-H1 expression compared to the same strain treated with CS obtained from the *agr*-knockout strain, mNY19335. Since these experiments were conducted during the stationary phase—when *ardA*-H1 transcript levels are elevated—the concentration of homologous AIP is also higher, providing strong competition against the heterologous AIP. As a result, the inhibition of *agr*-RNAIII was relatively modest, although it affected *ardA*-H1 expression (Figure 2a,b). To further validate these findings, we assessed *ardA*-H1 expression in the strain GV69, which is naturally deficient in RNAIII. Our result demonstrated that BMB9393 exhibited higher levels of *ardA*-H1 transcripts compared to GV69, with corresponding increases in *rnaIII* expression of 61.17-fold and 20.97-fold, respectively (Figure 2c,d).

### 2.3. Acidic Stress Drives ardA-H1 Expression in Strain BMB9393, While Temperature Shifts Show No Notable Effect

**Figure 3:**
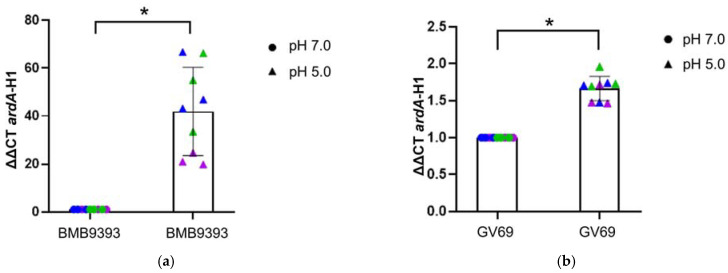

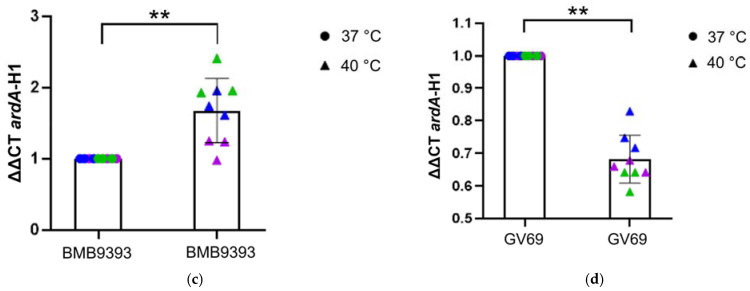
Relative quantitation (ΔΔCT) of *ardA*-H1 transcripts under acidic and temperature stresses. Panels (**a**,**b**) show strain BMB9393 and GV69, respectively, grown at pH 5.0 and pH 7.0. The data used for statistical analysis represent the mean of three independent biological experiments, each with three technical replicates (*n* = 3). A paired Student’s *t*-test was used to calculate the *p*-value, with significance indicated as (**a**) * for *p* = 0.0112 and (**b**) * for *p* = 0.0124. The corresponding Bayes’s factors were 1.055 for BMB9393 and 14.565 for GV69, supporting the alternative hypothesis over the null. Panels (**c**,**d**) display strains BMB9393 and GV69 grown at 37 °C and 40 °C, respectively. The data used for statistical analysis represent the mean of three independent biological experiments, each with three technical replicates *(n* = 3). A paired Student’s *t*-test was used to calculate *p*-value, indicated as (**c**) ** for *p* = 0.0036 and (**d**) ** for *p* = 0.0023. The corresponding Bayes factors were 6.731 for BMB9393 and 8.332 for GV69, further supporting the alternative hypothesis over the null. Individual data points from different replicates are represented in blue, green, and violet.

In strain BMB9393, *ardA*-H1 transcript levels increased 41.89-fold under acidic pH compared to neutral pH. In contrast, strain GV69, which exhibits attenuation in *agr*-RNAIII expression, showed a more modest 1.66-fold increase (Figure 3a,b). When exposed to 40 °C, the *ardA*-H1 transcripts in BMB9393 increased by 1.67-fold compared to 37 °C, whereas *ardA*-H1 transcripts in GV69 strain exhibited a 0.682-fold decrease in expression under the same condition (Figure 3c,d).

### 2.4. Iron-Depletion Stress Had an Important Positive Impact on ardA-H1 Expression in Strain BMB9393

**Figure 4:**
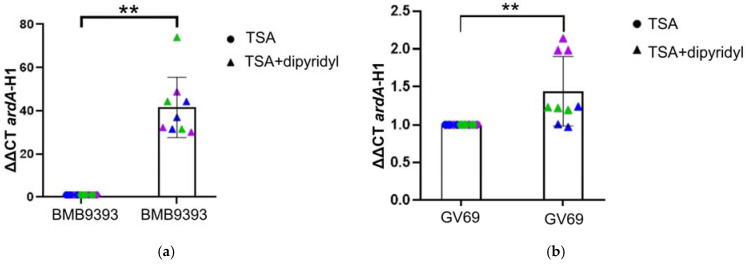
Iron depletion stress. Panels (**a**,**b**) show the strains BMB9393 and GV69, respectively, grown in tryptic soy agar (TSA) supplemented or not with 0.5 mM 2,2-dipyridyl. Individual data points from different replicates are represented in blue, green, and violet. The data used for statistical analysis represent the mean of three independent biological experiments, each with three technical replicates (*n* = 3). A paired Student’s *t*-test was used to calculate the *p*-value, with significance indicated as (**a**) ** for *p* = 0.0061 and (**b**) ** for *p* = 0.0031. The corresponding Bayes factors were 1.071 for BMB9393 and 6.696 for GV69, supporting the alternative hypothesis over the null.

Strain BMB9393, when grown with 2,2-dipyridyl, exhibited a high impact on *ardA*-H1 expression, displaying a 41.55-fold increase in *ardA*-H1 transcripts compared to normal culture conditions (Figure 4a). In contrast, the GV69 strain under iron-depletion conditions showed a more modest 1.44-fold increase in *ardA*-H1 transcripts compared to normal conditions (Figure 4b).

### 2.5. Biofilm Growth and Stressed Caused by Vancomycin Also Impacted ardA-H1 Expression

**Figure 5:**
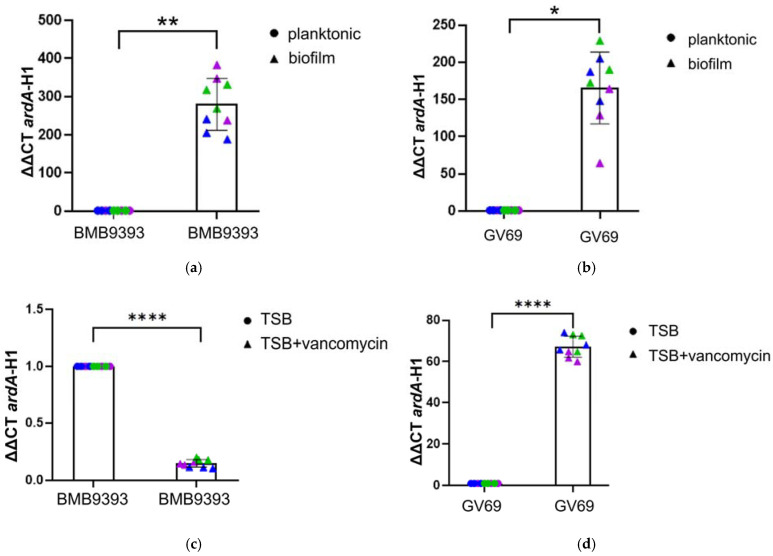
Effect of biofilm growth and exposure to 1/4 MIC of vancomycin on *ardA*-H1 expression. Panels (**a**,**b**) show the expression in BMB9393 and GV69, respectively, under biofilm and planktonic growth conditions. The data used for statistical analysis represent the mean of three independent biological experiments, each with three technical replicates (*n* = 3). A paired Student’s *t*-test was used to calculate *p*-value, with significance indicated as (**a**) ** for *p* = 0.0058 and (**b**) * for *p* = 0.0127. The corresponding Bayes factors were 1.010 for BMB9393 and 1.016 for GV69, supporting the alternative hypothesis over the null. Panels (**c**,**d**) display *ardA*-H1 expression in BMB9393 and GV69, respectively, under treatment with 1/4 MIC vancomycin. The data represent the mean of three independent biological experiments, each with three technical replicates (*n* = 3). A paired Student’s *t*-test was used to calculate the *p*-value, with significance indicated as (**c**,**d**) **** for *p* < 0.0001. The corresponding Bayes factors were 1.046 for BMB9393 and 1.472 for GV69, further supporting the alternative hypothesis over the null. Individual data points from different replicates are represented in blue, green, and violet.

BMB9393 sessile (biofilm) cells exhibited a 279.8-fold increase in *ardA*-H1 transcripts compared to planktonic cells (Figure 5a). Similarly, GV69 sessile cells, though showing a smaller increase than BMB9393, still displayed an important 165.5-fold rise in *ardA*-H1 expression (Figure 5b). Under the experimental conditions, the BMB9393 strain formed strong biofilms (BU = 2.66), while the GV69 strain produced moderate biofilms (BU = 0.86). An unexpected result was observed for strains treated with 1/4 of the minimal inhibitory concentration (MIC) of vancomycin. Despite the GV69 strain having an impaired Agr system, it showed a 67.24-fold increase in *ardA*-H1 transcripts compared to growth in media without antibiotics. In contrast, the Agr-functional BMB9393 strain exhibited a 7-fold decrease in *ardA*-H1 expression under the same condition (Figure 5c,d).

## 3. Discussion

The observed increase in *ardA*-H1 expression during the stationary phase suggests a temporal regulatory mechanism linked to Agr activation. Agr is a quorum sensing system so its activity initiates only when AIP accumulates extracellularly, which typically occurs from the mid-exponential phase to the stationary phase [23]. Since Agr regulates not only virulence factors but also genes essential for survival under nutrient-limited conditions—such as those activated during the stationary phase and biofilm growth [24]—it is plausible that *ardA*-H1 is part of this adaptive response for survival. The associated increase in HGT during this phase may enhance the bacterial ability to acquire beneficial extracellular DNA (eDNA), thereby improving its adaptability under selective pressure.

Previous studies have demonstrated that CS containing heterologous AIP (i.e., an AIP of a different type than that produced by the tested strain) can inhibit the expression of the Agr system in the test strain when added to the culture medium [23,25]. Supporting the hypothesis that *ardA*-H1 is under the regulatory control of the Agr system, inhibition of RNAIII transcripts by heterologous AIP led to a reduction in *ardA*-H1 expression. Furthermore, the GV69 strain, which has a natural Agr attenuation, also exhibited reduced RNAIII transcript levels, which correlated with lower *ardA*-H1 expression.

An important increase in *ardA*-H1 expression was observed in the Agr-functional strain BMB9393 under acidic stress, compared to the Agr-dysfunctional GV69 strain. In GV69, *ardA*-H1 expression exhibited a modest increase of less than 2-fold. Studies by Weinrick et al. showed that *agr* is induced at pH 5.5 [26]. Therefore, these findings suggest that the increased *ardA*-H1 expression in BMB9393 is dependent on Agr function.

It is well known that both acidic and oxidative stresses can activate overlapping cellular pathways and stress responses. For example, oxidative stress can induce the expression of genes that contribute to maintaining pH balance, thereby influencing the response to acidic stress. Conversely, acidic pH can increase hydrogen peroxide (H_2_O_2_) levels, further intensifying oxidative stress. The Agr system integrates bacterial metabolism with virulence and environmental adaptation, particularly by boosting bacterial survival under lethal concentrations of H_2_O_2_, a key component of host defense against *S. aureus* and other bacteria. Notably, ROS accumulated more extensively in the *agr* mutant than in wild-type (WT) cells, thereby explaining the increased susceptibility of *agr*-knockout strains (Δ*agr*) to lethal doses of H_2_O_2_ and evidencing the role of Agr as an important global virulence regulator that also responds to oxidative stress [27]. Importantly, the increase in *ardA*-H1 expression under acidic conditions has the potential to enhance HGT, as *ardA*-H1 expression in different *S. aureus* backgrounds has been shown to enhance the gene transfer rate [19].

It is well established that during the stationary growth phase, bacterial cells face adverse environmental conditions, leading to an increase in cell death. This rise can be attributed to the accumulation of toxic metabolites, medium acidification, and oxidative stress [28,29]. To counter these challenges, bacteria elevate their mutation rates, facilitating adaptation to hostile environments—a crucial strategy for survival amidst the fluctuating conditions frequently encountered in nature [30]. Indeed, oxidative stress triggered by a sudden increase in H_2_O_2_ concentration has been shown to cause a rapid rise in mutation rates, as measured in *E. coli* in vitro [31]. In addition, the increase in ROS induced by various stressors may also enhance horizontal gene transfer (HGT) through mechanisms such as the upregulation of genes associated with the SOS response system [10,11].

The observed increase in *ardA*-H1 expression under acidic conditions supports the idea of Agr-dependent regulation, which may enhance bacterial diversification when exposed to such adverse and toxic environments. Therefore, it is reasonable to hypothesize that increased *ardA*-H1 expression during the stationary growth phase and/or under acidic conditions may facilitate the incorporation of new genetic elements through HGT, providing a potential advantage to bacteria facing these challenges. Notably, human tissues, such as the skin, exhibit acidic pH, ranging from 4.5 to 5.5. Similarly, extracellular acidity is also present in inflamed tissue and the microenvironment of tumors [32].

No important impact on *ardA*-H1 expression was observed in either BMB9393 or GV69 when the growth temperature was shifted from 37 °C to 40 °C. Although the *p*-value was significant, the differences in *ardA*-H1 expression between the two temperatures did not exceed 2-fold in either strain. *S. aureus* responds to heat stress through various regulatory systems; however, to the best of our knowledge, no studies have associated the Agr quorum sensing system with the heat stress response.

In contrast, a substantial impact on *ardA*-H1 expression was observed in BMB9393 but not in the Agr-deficient GV69 under iron-restricted conditions. These data further support the hypothesis that Agr is an important positive regulator of *ardA*-H1, as *agr* expression is known to increase under iron restriction [33]. Studies by Allard et al. investigated the global gene expression under normal iron-restricted in vivo conditions and under in vitro iron deprivation with 2,2-dipyridyl. They demonstrated that *agr*-RNAIII was not only upregulated in vivo but also that various Agr-regulated genes were upregulated in vitro [34].

Fur (ferric uptake regulator) is a transcriptional regulator essential in genes involved with iron homeostasis. Additionally, Fur plays a prominent role in *S. aureus* virulence by interacting with important virulence regulators including Agr. These regulators modulate bacterial physiology and virulence, in response to iron availability, by controlling the expression of hemolysins, adhesins, and leucocidins, and facilitating biofilm formation [33]. Despite the fact that the complexity of this signaling network remains not fully understood [35], these findings are relevant given the limited availability of iron in the human host, as most of them are in a complex with transferrin, lactoferrin, and heme groups [36]. Collectively, these results implicate Agr quorum-sensing in an iron-sensing network, contributing to the increased expression of *ardA*-H1 observed under iron restriction in the Agr-functional strain BMB9393.

Both BMB9393 and GV69 strains showed a remarkable increase in *ardA*-H1 expression under biofilm growth. Even though BMB9393 exhibited nearly double the increase observed in GV69, it suggests that other factors beyond *agr* may also contribute to this enhancement. Bacteria present within mature biofilms exhibit diverse and unique metabolic characteristics that enable them to resist harmful environmental stressors. These adverse effects arise from the microenvironment created by cell aggregation, leading to an accumulation of toxic metabolites, decreased nutrient availability, and low oxygen levels in the deeper layers of biofilms [37].

The Agr system is also an important regulator of biofilm production. In certain strains, the inactivation of Agr results in increased biofilm production. However, in strains of the ST239-SCC*mec*III lineage (such as BMB9393 and GV69 strains), the inactivation of Agr decreases biofilm production, indicating a positive regulatory role of Agr. This discrepancy can be attributed to the role played by Agr as a global regulator of virulence and the multifactorial composition of biofilms, as their matrix may vary among different *S. aureus* strains [6,38]. Consequently, the increased biofilm accumulation by the Agr-functional BMB9393 likely results in a higher presence of biofilm stressors and enhanced *ardA*-H1 expression compared to the GV69 strain, which forms less biofilm. This is significant since biofilm formation is a critical aspect of natural infections in host tissues, playing a major role in bacterial pathogenesis and infection persistence.

The higher cell density in biofilms may also promote an increase in eDNA through cell lysis, which could, in turn, facilitate HGT and the acquisition of MGE. Maree et al. observed the transfer of SCC*mec* elements from MRSA strains, as well as from methicillin-resistant coagulase-negative staphylococci to MSSA strains within biofilms. They also demonstrated that, when cell growth occurs in biofilms, two-component regulatory systems, including Agr, regulate the increased expression of natural competence genes in *S. aureus* [39]. Therefore, our data show an expressive increase in *ardA*-H1 transcripts in biofilms, which introduces an additional factor that may possibly enhance the acquisition of resistance and virulence genes. These findings also support the observation that *S. aureus* strains carrying *ardA*-H1 exhibit a higher rate of multidrug resistance compared to *ardA*-negative strains [19].

One of the main environmental challenges faced by pathogenic bacteria during infections is the presence of antimicrobials. Vancomycin, one of the last resort treatments for serious MRSA infections, has been shown in both in vitro and in vivo studies to enhance cell growth and biofilm production at sub-minimal inhibitory concentrations (sub-MIC) [40]. A study demonstrated that exposure to sub-MIC vancomycin led the *agr*-negative *S. aureus* strain RN9120 to develop an autolysin-deficient phenotype, whereas the isogenic wild-type strain RN6607 exhibited only minimal changes. Moreover, the same study reported that the *agr*-knockout strain displayed increased susceptibility to vancomycin compared to its isogenic WT counterpart [41]. These findings align with our observation that the response of *ardA*-H1 expression to vancomycin differed importantly between GV69 and BMB9393. While *ardA*-H1 expression increased approximately 67-fold in GV69, a 7-fold decrease was observed in the Agr-functional BMB9393 strain. Interestingly, Agr-deficient strains, such as GV69 in our investigation, can naturally emerge during infections [42].

Our results suggest that other mechanisms are involved in the vancomycin activation of *ardA*-H1 in the Agr-deficient strain indicating the involvement of alternative regulatory pathways. Additionally, the findings support the notion that Agr-deficient *S. aureus* strains possess an intrinsic survival advantage under vancomycin selective pressure [41]. Beyond mere survival, these strains may also exploit such stress conditions to activate their evolutionary triggering mechanisms, thereby accelerating adaptation through the engagement of the AR system.

## 4. Study Limitation

A limitation of this study is that we used only two strains of the ST239-SCC*mec*III lineage, which may not fully capture the variability in *ardA*-H1 expression across different strain backgrounds. It is possible that other strain lineages could respond differently to stressors in relation to *ardA*-H1 expression. Although the *ardA*-H1 gene is distributed among various Gram-positive bacteria, in *S*. *aureus*, it is predominantly found among highly multidrug-resistant strains belonging to ST239-SCC*mec*III, with very rare occurrences in other lineages [19].

Another limitation is the absence of an *agr* knockout within the ST239-SCC*mec*III background. Cloning strategies in *S*. *aureus* are notoriously challenging, particularly in ST239 strains, which exhibit resistance to most commercial antimicrobials with the exception of vancomycin and other newer anti-MRSA agents. To address this, we employed the supernatant from a heterologous type II AIP-producing strain to inhibit Agr type I activity in the BMB9393 strain. Additionally, we included an Agr*-*deficient ST239 strain GV69, which clusters within the same major phylogenetic clade as the Agr-functional strain BMB9393 [43], to provide further insights into *ardA*-H1 expression under these conditions.

Finally, while we did not conduct specific studies to determine whether the enhanced expression of *ardA*-H1 promoted by stress conditions would result in an increased rate of HGT, previous research from our group demonstrated that the expression of *ardA*-H1 cloned into an *ardA*-negative strain was enhanced more than threefold across different *S*. *aureus* backgrounds, along with successful heterologous expression in an *E*. *coli* strain [19].

## 5. Materials and Methods

### 5.1. Bacterial Strains

For gene expression experiments, we utilized the MRSA strain BMB9393, a highly multiresistant nosocomial bacterium isolated from a bloodstream infection. This strain is *agr*-positive type I (functional) and harbors *ardA*-H1 [19,42]. We also included MRSA strain GV69, a skin wound isolate, which is *ardA*-H1 positive but has a dysfunctional type I Agr system [19,43]. Both BMB9393 and GV69 were typed as ST239-SCC*mec*III and belong to the Brazilian epidemic clone (BEC). For the preparation of CS containing the heterologous Agr type II peptide, we utilized the isogenic WT strain NY19335 (ST5-SCC*mec*II; Agr functional) and the *agr*-knockout strain mNY19335 as a negative control [32]. The strains used in this study are listed in Table 1.

### 5.2. Expression of ardA-H1 at Exponential and Stationary Phases

An 18-h culture of the BMB9393 strain was diluted 1:1000 in tryptic soy broth (TSB; GIBCO, Waltham, MA, USA) and incubated at 37 °C with shaking at 250 rpm, until the culture reached an optical density (OD) of 0.3 at 600 nm, corresponding to the beginning of the exponential phase. For the stationary phase, bacteria were diluted 1:100 in TSB and incubated under the same condition until reaching an OD of 2.2. The culture was then immediately mixed with an equal volume of a cold acetone-ethanol solution (1:1; *v*/*v*) to inhibit nucleases and stored at −80 °C until RNA preparation, as described below. All samples were tested in biological triplicates, with three technical replicates for each. These OD were chosen because at OD 0.3, the amount of *agr*-RNAIII is very low compared to its level at OD 2.2 [23].

### 5.3. Role of Agr in ardA-H1 Regulation

A strategy employed to inhibit Agr system in this study involved the use of CS that either contained or did not contain the AgrA autopeptide II. Since this autopeptide is heterologous to the Agr type I of the BMB9393 strain, it inhibits Agr activity in this strain [23,38]. To prepare CS, an 18-h culture of the isogenic strains NY19335 (WT) or mNY19335 (∆*agr*) was diluted 1:100 in TSB and incubated at 37 °C with shaking at 250 rpm for 18 h. The cultures were then sterilized by filtration using a 0.22 µm membrane (JET Biofil, Guangzhou, GD, China). The CS was aliquoted and stored at −80 °C until use. An 18-h culture of the strain BMB9393 was diluted 1:100 in TSB supplemented with 10% CS prepared from either NY19335 or mNY19335 and then the bacterial suspensions were incubated for 18 h at 37 °C with shaking at 250 rpm, until reaching an OD of 2.2 at 600 nm. The culture was then immediately mixed with an equal volume of a cold acetone-ethanol solution (1:1; *v*/*v*) and stored at −80 °C until RNA preparation [38]. All samples were prepared in biological triplicates, with three technical replicates for each.

Another strategy to investigate the role of Agr in regulating *ardA*-H1 was to assess the expression of the *ardA*-H1 gene in the Agr-functional strain BMB9393 and the Agr-dysfunctional strain GV69 [42,43]. To this end, an 18-h culture of each strain was diluted 1:100 in TSB and incubated for 18 h at 37 °C with shaking at 250 rpm, until reaching an OD of 2.2 at 600 nm. Each culture was then immediately mixed with an equal volume of a cold acetone-ethanol solution (1:1; *v*/*v*) and stored at −80 °C until RNA preparation. All samples were prepared in biological triplicates, with three technical replicates for each.

### 5.4. Effect of Acidic pH on ardA-H1 Expression

An 18-h culture of strains BMB9393 or GV69 was diluted 1:100 in TSB pH 7.0 or TSB pH 5.0, acidified with 1.0 M HCl (Merck, Darmstadt, HE, Germany) [44] and incubated for 18 h at 37 °C with shaking at 250 rpm, until reaching an OD of 2.2 at 600 nm. A cold acetone-ethanol solution (1:1; *v*/*v*) was immediately added to the cultures, which were frozen at −80 °C until RNA preparation. All samples were prepared in biological triplicates, with three technical replicates for each.

### 5.5. Role of Iron Deprivation in ardA-H1 Expression

An inoculum from an 18-h culture of strain BMB9393 or GV69 was spread on tryptic soy agar (TSA; GIBCO) with or without a final concentration of 0.5 mM of 2,2-dipyridyl (Sigma-Aldrich, Saint Louis, MO, USA) [34,45], to reach confluent growth and incubated at 37 °C for 18 h. The cells were collected in TE buffer solution (Tris-HCl 10 mM; EDTA 1 mM pH 8), and an equal volume of a cold acetone-ethanol 1:1 (*v*/*v*) mixture was added to the samples, which were frozen at −80 °C until RNA preparation. All samples were tested in biological triplicates, with three technical replicates for each.

### 5.6. Bacterial Growth Under Biofilm Condition

For biofilm formation, BMB9393 or GV69 strains were streaked on TSA and incubated for 24 h at 37 °C. Approximately three colonies of each strain were then inoculated in TSB supplemented with 1% (*w*/*v*) glucose (TSB-Glu) and incubated for 24 h at 37 °C with shaking at 250 rpm. The cells were diluted (1:100) in TSB-Glu, and 200 μL aliquots were added into each well of a sterile 96-well plate (Nunclon untreated; Nunc A/S, Roskilde, Zl, Denmark) for 20-h incubation at 37 °C. The absorbance was measured at 570 nm (A1) using a SpectraMax Plus 384 Absorbance Microplate (Molecular Devices, Sunnyvale, CA, USA). The wells were washed with nuclease-free water to remove non-adherent bacteria. Biofilms were scraped off using a sterile toothpick, treated with an equal volume of cold acetone ethanol solution (1:1), and stored at −80 °C. In parallel, additional plates containing biofilms were stained with crystal violet to quantify the biofilms, with a spectrophotometric reading at 570 nm (A2). Biofilm units were calculated using the formula: BU = A2/A1. The isolates were classified as non-producers (BU ≤ 0.230), weak producers (BU > 0.230 and <0.460), moderate producers (BU > 0.460 and <0.920), or strong producers (BU ≥ 0.920) [46]. For planktonic cell preparation, an 18-h culture of bacterial strains BMB9393 or GV69 was diluted in TSB-Glu 1:100 and incubated for 18 h at 37 °C with shaking at 250 rpm, until reaching an OD of 2.2 at 600 nm. An equal volume of cold acetone-ethanol 1:1 (*v*/*v*) solution was added to the samples, which were then stored at −80 °C until RNA preparation. All samples were tested in biological triplicates, with three technical replicates for each.

### 5.7. The Effect of Vancomycin on ardA-H1 Expression

An 18-h culture of the strain BMB9393 or GV69 was diluted (1:100) in TSB with or without 0.5 µg/mL of vancomycin (Sigma-Aldrich), which corresponds to 1/4 MIC [47]. Cultures were incubated at 37 °C for 18 h with shaking at 250 rpm, until reaching an OD of 2.2 at 600 nm. An equal volume of a cold 1:1 (*v*/*v*) acetone-ethanol solution (Merck) was added to the cultures. The mixture was then stored at −80 °C until RNA preparation. All samples were tested in biological triplicates, with three technical replicates for each.

### 5.8. RNA Preparation

Frozen bacterial cell suspension in acetone-ethanol solution was thawed and centrifuged at 8700× *g* at 4 °C. The pellet was washed twice with TE buffer, using half the original volume of cell suspension, and centrifuged again. The bacteria were then resuspended in 10 μL of solution of 10 U/μL lysostaphin and 90 μL of TE buffer. The mixture was incubated on ice for 30 min, followed by 5-min incubation at 37 °C [48]. After this procedure, the RNA was obtained according to the RNeasy Mini Kit protocol (Qiagen, Hilden, DE, Germany). The quantity of obtained RNA was measured using a Nanodrop spectrophotometer (Thermo Fisher Scientific, Waltham, MA, USA).

The integrity and quality of the total RNA were verified through gel electrophoresis on a 1.2% (*w*/*v*) agarose gel in TAE buffer (20 mM Tris acetate, 0.5 mM EDTA pH 8.0), stained with 5 μg/mL ethidium bromide and visualized under ultraviolet (UV) light using the GelDoc™ EZ Imager (BioRad, Hercules, CA, USA).

Subsequently, 1 µg of RNA was treated with 2 U DNase I Amplification Grade (Invitrogen, Carlsbad, CA, USA) and incubated at 25 °C for 20 min in a thermocycler (Veriti™ 96-Well Thermal Cycler, Applied Biosystems, Foster City, CA, USA). The reaction was terminated by adding 25 mM EDTA (pH 8.0) and heating at 65 °C for 10 min. After treatment, the RNA was quantified with a Nanodrop spectrophotometer (Thermo Fisher Scientific) and stored at −80 °C.

Finaly, a PCR reaction mixture was prepared to verify the absence of RNA contamination with DNA. To this end, 5 μL of GoTaq DNA polymerase (Promega, Madison, WI, USA), 0.2 μM of each *mecA* primer, forward and reverse, and 2 μL of RNA were added to sterile nuclease-free water, bringing the total volume to 10 μL. A negative control was prepared using water instead of RNA, while a positive control was included with MRSA DNA from strain CD16-182 from ST105(CC5)-SCC*mec*II. The reaction was conducted in a thermocycler (Veriti™ 96-Well Thermal Cycler, Applied Biosystems), under the following conditions: 94 °C for 4 min; followed by 30 cycles of denaturation at 94 °C for 30 s, annealing at 53 °C for 30 s, and extension 72 °C for 1 min; concluding with a final extension at 72 °C for 4 min. The PCR products were analyzed by electrophoresis on a 2.0% agarose gel in TAE buffer, stained with ethidium bromide, and visualized using the GelDoc™ EZ Imager (BioRad). The primers used to amplify the *mecA* gene are listed in Table 2.

### 5.9. Real Time qRT-PCR

Transcripts were quantified using real-time qRT-PCR, employing the ΔΔCT methodology and Power SYBR Green RNA-to-CT™ 1-Step kit (Applied Biosystems) according to the manufacturer’s instructions on a Step One Real-Time PCR System platform (Applied Biosystems). The transcripts of *rrs* encoding 16 S rRNA were used as an endogenous control. The results were normalized, assigning a value of 1.0 to the calibration sample. The primers used for *ardA*-H1, *agr*-RNAIII, and *rrs* are listed in Table 2.

### 5.10. Statistical Analyses

Both paired and unpaired two-tailed Student’s *t*-tests were employed, depending on the experiment, to quantify differences in gene expression between test and control samples across three independent biological experiments, each with three technical replicates, using GraphPad Prism 9 software. Furthermore, Bayes’ Factor was calculated [51], to challenge the alternative hypothesis against the null hypothesis, utilizing the Bayes Factor Calculator (https://medstats.github.io/bayesfactor.html (accessed on 18 October 2024).

## 6. Conclusions

Our findings highlight an important interplay between environmental stressors, quorum sensing regulation, and *ardA*-H1 expression in *S*. *aureus*. While the Agr system appears to modulate *ardA*-H1 expression under conditions such as acidic pH, iron limitation, and biofilm growth, its activation by vancomycin also seems to proceed independently of *agr*, suggesting the involvement of alternative regulatory pathways. This flexible regulation may promote HGT, potentially enhancing the bacterial ability to acquire beneficial traits.

The differential responses observed in Agr-deficient strain GV69 under vancomycin exposure indicate that the inhibition of quorum sensing activity may confer certain advantages, possibly by limiting autolysis, increasing persistence, as suggested previously, and preserving genetic adaptability.

To the best of our knowledge, this is the first study to demonstrate the impact of stressors—encountered during natural infectious processes—on boosting the expression of an AR gene. Although further studies are necessary to fully elucidate these mechanisms, our data align with the idea that *S*. *aureus* navigates environmental stresses not only to ensure survival but also to foster diversification, underscoring the role of quorum sensing and HGT in shaping its persistence and adaptability.

Understanding the molecular and evolutionary pathways that enable bacteria to enhance gene transfer is crucial for the development of next-generation antimicrobials. Future therapeutics should aim not only to eradicate bacterial pathogens but also to target and disrupt the cellular mechanisms that foster genetic adaptability and the emergence of multidrug-resistant mutants.

## Figures and Tables

**Table 1:** *Staphylococcus aureus* strains were used in the current study.

Strain [Reference]	Lineage and Genetic Characteristics
BMB9393 [19,42]	ST239-SCC*mec*III, *ardA*-H1, type I *agr* (Agr functional)
GV69 [19,43]	ST239-SCC*mec*III, ardA-H1, type I *agr* (Agr dysfunctional)
NY19335 [38]	ST5-SCC*mec*II, type II *agr* (Agr functional)
mNY19335 [38]	ST5-SCC*mec*II, Δ*agr* (derived from NY19335)

**Table 2:** RT-qPCR primers.

Primer	Direction	Sequence (5′-3′)	Reference
*mecA*	Forward	AGATGATACCTTCGTTCCACT	[49]
	Reverse	CTGGTGAAGTTGTAATCTGGA	
*ardA*-H1	Forward	CGTCTATGTGAAGCCATTCAA	[19]
	Reverse	TTCCAGTAATTCTTCCAAGCTA	
*rnaIII*	Forward	ATGATCACAGAGATGTGA	[50]
	Reverse	CTGAGTCCTAGGAAACTAACTC	
*rrs*	Forward	AGAGATAGAGCCTTCCCCTT	[46]
	Reverse	TTAACCCAACATCTCACGACA	

## Data Availability

The original contributions presented in the study are included in the article; further inquiries can be directed to the corresponding author.
